# Correction: Dearomative logic in natural product total synthesis

**DOI:** 10.1039/d2np90039d

**Published:** 2022-11-22

**Authors:** Christopher J. Huck, Yaroslav D. Boyko, David Sarlah

**Affiliations:** Department of Chemistry, University of Illinois Urbana IL 61801 USA sarlah@illinois.edu; Department of Chemistry, University of Pavia Viale Taramelli 12 27100 Pavia Italy

## Abstract

Correction for ‘Dearomative logic in natural product total synthesis’ by Christopher J. Huck *et al.*, *Nat. Prod. Rep.*, 2022, https://doi.org/10.1039/d2np00042c.

The authors regret that the stereochemistry of intermediates 270 and 271 (Fig. 31) is incorrect in the article. The correct structures and Fig. 31 are displayed below.

**Fig. 31:**
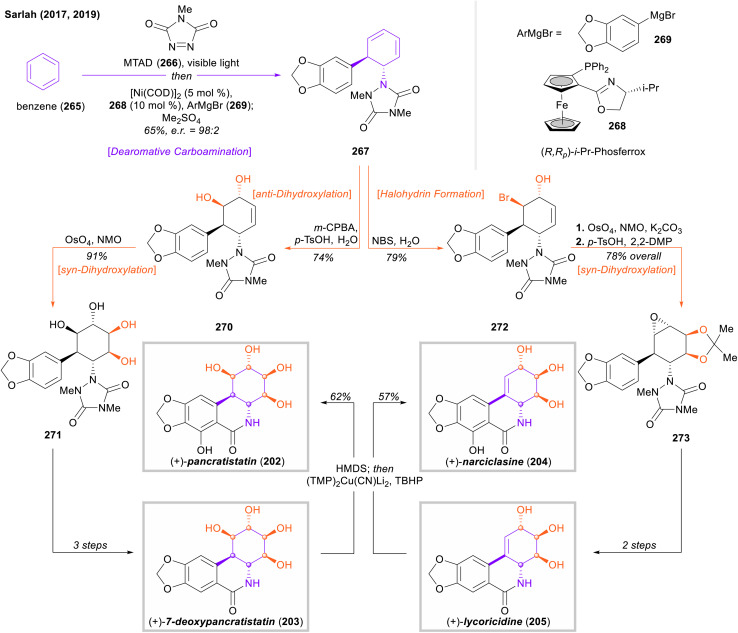
Sarlah's catalytic desymmetrization of benzene and application to the total synthesis of pancratistatin, 7-deoxypancaratistatin, narciclasine, and lycoricidine.

The Royal Society of Chemistry apologises for these errors and any consequent inconvenience to authors and readers.

## Supplementary Material

